# TNF gene deletion prevents lipopolysaccharide-mediated sensitisation of the neonatal mouse brain to hypoxic-ischaemic insult

**DOI:** 10.1186/1753-6561-6-S4-O9

**Published:** 2012-07-09

**Authors:** A Sahota, G Kendall, S Lange, G Raivich

**Affiliations:** 1 Perinatal Brain Repair Group, Institute for Women's Health, University College London, UK

## Introduction

An increasing body of evidence suggests a synergistic link between infection/inflammation and hypoxia-ischaemia in the pathogenesis of perinatal brain injury. Deletion of the TNF cytokine gene cluster (TNF, LTα and LTβ) has previously been shown to abolish lipopolysaccharide (LPS)-mediated sensitisation of the developing brain to hypoxic-ischaemic (HI) insult. In this study, I investigated if single TNF and LTβ gene deletions prevented LPS-sensitised HI brain injury.

## Methods

Postnatal day 7 mice homozygous for either TNF or LTβ cytokine gene deletions received either 0.3mcg/g LPS or saline by intraperitoneal injection 12 hours prior to 30-minute HI insult. Coronal forebrain sections were examined for brain injury using Nissl stain and microglial activation using the activation marker αMβ2 intergrin (αM). Injury was scored in ipsilateral grey matter regions and external capsule white matter (ipsilateral and contralateral). Values given are mean ± SEM and data was analysed for significant differences using unpaired two-tailed Student's t-test.

## Results

Pre-treatment with LPS in wild-type mice (n=13) resulted in significantly increased overall brain injury (0.96±0.17 v 3.11±0.44, p < 0.05) and αM expression across all assessed ipsilateral forebrain regions (p < 0.05) compared to saline pre-treated controls (n=13). TNF knockout animals pre-treated with LPS (n=14) did not show a significant difference in overall brain injury (2.82±0.50 v 2.90±0.57, p = 0.91) or regional αM expression compared to saline controls (n=13). Wild-type animals from the LTβ breeding group did not exhibit increased overall brain injury in response to LPS pre-treatment (n=4) compared to saline controls (n=4).

## Conclusions

Deletion of the TNF cytokine gene prevents lipopolysaccharide-mediated sensitisation of the neonatal brain to hypoxic-ischaemic insult. Sensitisation to LPS was not seen in the wild-type animals in the LTβ strain, which could suggest possible spontaneous mutation of the gene(s) responsible for LPS response in the strain used. Future work into the effects of LTα and LTβ gene deletions, cell-specific gene deletions and pharmacological inhibition of cytokines may help further the understanding of the mechanisms involved in endotoxin-sensitised HI brain injury and provide possible therapeutic options to minimise injury to the developing nervous system.

